# Estimating malaria transmission in Sarangani Province, the Philippines using serological markers of infection

**DOI:** 10.1186/1475-2875-11-S1-P20

**Published:** 2012-10-15

**Authors:** Mary Grace Dacuma, Judeline Dimalibot, Federico Yadao, Antonio Yasaña, Ernesto Bona, Walter Notario, Rachel Hallett

**Affiliations:** 1 London School of Hygiene and Tropical Medicine, London, WC1E 7HT, UK; 2 University of the Philippines Los Baños, Laguna, 4031, Philippines; 3 Provincial Health Office, Alabel, Sarangani Province, 9501, Philippines; 4 Department of Health Region XII, Alabel, Sarangani Province, 9501, Philippines; 5 Pilipinas Shell Foundation, Alabel, Sarangani Province, 9501, Philippines

## Background

The Philippines is among the 39 countries aiming for malaria elimination. The major challenge is finding residual transmission foci in difficult to access villages of Southern Mindanao, Philippines. The sensitivity of microscopy to detect asymptomatic infections declines with decreasing malaria prevalence. The aim of this project is to use antibody markers of *P. falciparum* and *P. vivax* infection to locate residual transmission foci and to determine effects of control measures in Sarangani Province, the Philippines.

## Methods

Filter paper blood spots from 907 participants (aged 1 - 86 yrs) were collected in a cross-sectional survey from nine villages in Sarangani Province, Philippines between June - August 2010. Sera extracted blood spots were tested for presence of antibodies to *P. falciparum* and *P. vivax* apical membrane antigen 1 (AMA1) and merozoite surface protein 1 (MSP1_19_) using indirect ELISA [1].The mixture model was used to define the cut off value for presence or absence of antibody to the antigen tested. The dichotomized sera were fitted into reversible prevalence catalytic models using maximum likelihood estimation to generate age-specific seroprevalence curve [2]. Statistical analyses were done using the STATA^®^ v12 software (Stata Corp., Texas).

## Results

The age-specific seroprevalence curves for Pf-AMA1 and Pf-MSP1_19_ showed that the force of *P. falciparum* infection (λ) per year in Sarangani Province was 0.005 (95% CI: 0.003 - 0.009) and 0.03 (95% CI: 0.02 - 0.05), respectively. This result was supported by records of annual parasite incidence (API per 1,000) in Sarangani that decreased from 3.49 in 2005 to 0.57 in 2009. There was strong evidence (P < 0.001) that the difference in exposure to *P. falciparum* varies between the nine villages. The age-specific seroprevalence curve to Pv-AMAI showed that in 2010 the force of *P. vivax* infection (λ) per year was 0.02 (95% CI: 0.01 - 0.04), which reflected a decline in transmission as observed in seroprevalence curves to Pf-AMA1 and Pf-MSP_19_.The force of infection using Pv-MSP_19_ was not calculated because its seroprevalence curve showed a horizontal line (probability positive of 0.1). This suggested that either *P. vivax* epidemic occurred in the province where individuals exposed developed antibodies to *P. vivax* at one point in time as observed in Vanuatu [3]; or a relapse of *P. vivax* infections due to activation of liver hypnozoites. There was strong evidence (P < 0.001) that *P. vivax* transmission differed between the villages surveyed.

## Conclusion

The results show that *P. falciparum* and *P.vivax* transmission continue at very low levels in Sarangani Province, the Philippines despite strengthened control efforts. It is recommended that elimination efforts are intensified in villages where antibody prevalence to *P. falciparum* and *P. vivax* infections is higher relative to the nine villages surveyed.
